# An Innovative Treatment Approach Using Digital Workflow and CAD-CAM Part 1: The Restoration of Endodontically Treated Molars in Children

**DOI:** 10.3390/ijerph17041364

**Published:** 2020-02-20

**Authors:** Esti Davidovich, Boaz Shay, Eyal Nuni, Eitan Mijiritsky

**Affiliations:** 1 Department of Pediatric Dentistry, Hebrew University, Hadassah School of Dental Medicine, Jerusalem 91120, Israel; 2 Endodontic Department, Hebrew University, Hadassah School of Dental Medicine, Jerusalem 91120, Israel; boaz@endo.co.il (B.S.); clinic@drnuni.com (E.N.); 3 Department of Otolaryngology Head and Neck Surgery and Maxillofacial Surgery Tel-Aviv Sourasky Medical Center, Sackler School of Medicine, Tel Aviv University, Tel-Aviv 6139001, Israel; mijiritsky@bezeqint.net

**Keywords:** CAD-CAM, intraoral scanners, endocrowns, children

## Abstract

Stainless steel crowns are the most popular restoration technique for young permanent first molars treated endodontically. However, these restorations are not aesthetically appealing and need to be replaced. Endocrowns constitute a reliable approach for restoring severely damaged molars and premolars. Intraoral scanners (IOSs) are well tolerated by children and are easily and quickly implemented. We present an innovative treatment approach for endodontically treated teeth in children, using a digital workflow with IOS and computer-aided design/computer-assisted manufacturing (CAD/CAM) fabrication of the restoration. The protocol involves a thorough diagnostic phase and comprehensive treatment planning. Among the factors to be considered are the child’s behavior, the parents’ cooperation and compliance, and initial preparation including inhalation sedation, effective local anaesthesia and the use of a rubber dam. Full attention should be given to 1–2 mm of ferruling by the endocrown, which should be appropriately prepared to accommodate endocrowns for molars. IOSs include scanning of the prepared tooth and its antagonist, and scanning of the bite. CAD-CAM preparation of the restoration is followed by restoration bonding and follow up. Digital workflow should be considered in the treatment of endodontically treated molars since the high accuracy of the scanning enables definitive restoration in young patients.

## 1. Introduction

Several methods are available for restoring endodontically treated teeth, including the use of fixed partial dentures and various restorative materials [1]. A number of publications have recommended that following endodontic therapy, posterior teeth require adequate full-coverage restoration. This is to reduce the risk of fracture, to provide a coronal seal that avoids bacterial infection and to restore function [2,3].

In children, stainless steel crowns (SSCs) are the most popular mode of restoration for young permanent first molars treated endodontically. According to the guidelines of the American Academy of Pediatric Dentistry [4], metal crowns (SSCs) are indicated for treating permanent molars with extensive caries and developmental defects in children, following the failure of other available restorations, and for endodontically-treated teeth. The rationale for the use of SSCs is their cost-effectiveness, based on the durability and longevity of the restoration. SSCs protect teeth from future decay due to their full coverage, and their increased strength and durability [5].

At 24 months after restoration, preformed metal crowns and cast crowns placed on permanent teeth were shown to be similar in quality and longevity [6]. However, a Cochrane systematic review reported greater longevity for SSCs than for amalgam restoration [7]. Despite their advantages, SSCs have a few drawbacks. First, their metal appearance is generally considered unsatisfactory by both children and parents. Second, the preparation of SSCs requires obtaining dimensions of the entire tooth; this results in loss of tooth material. Third, SSCs for permanent molars are an interim restoration that must be replaced after adolescence by a full coverage crown.

Endocrowns were first introduced in 1995 by Patrik Pissis [8] as mono-block ceramic crowns bonded to endodontically-treated posterior teeth without the need for post and core retention. This minimally invasive approach is easily performed, less expensive, and most importantly, can decrease failures related to post-placement [9,10]. Belleflamme et al. examined 99 endocrown restorations over a mean period of 45 months [11]. Endocrowns were shown to constitute a reliable approach to restoring severely damaged molars and premolars, even when extensive coronal tissue is lost or occlusal risk factors present, such as bruxism and unfavorable occlusal relations. Moreover, a systematic review showed a 94%–100% success rate of endocrowns and higher fracture strength than with conventional treatments [12]. The authors suggested that endocrowns may perform similarly or better than conventional treatments that use intraarticular posts, direct composite resin, or inlay/onlay restorations.

Post and core procedures may be required in situations involving severe loss of coronal hard tissue. However, these may decrease tooth fracture resistance due to the need to remove additional dentin, and the increased risk of root perforation [1,13]. Yet, endocrowns can replace the need for post and core retention [11]. The development of adhesive dentistry has reduced the need for post and core retention to restore endodontically non-vital posterior teeth with extensive coronal tissue loss [1,12,13,14].

In children and adolescents, restoring endodontically-treated posterior molars with an aesthetic and long-term restoration is extremely difficult. This is primarily due to the challenge of obtaining the patient’s cooperation, especially during the impression-taking procedure, which is highly technique sensitive.

Intraoral scanners (IOSs) are powerful digital devices used for optical impressions. IOSs enable the collection of information on the shape and size of dental arches through the projection of a beam of light grid (structured light or laser) onto the tooth surface [15,16]. The scanners thereby capture, through high-resolution cameras, the distortion of the beam or grid when it reaches these structures [17]. The information collected by the cameras is processed using powerful software that reconstructs the three-dimensional (3D) model of the desired structures [16,17,18]. In particular, from the genesis of a “cloud of points”, a polygonal mesh is derived, which represents the scanned object. The scan is then processed to obtain the final 3D model [16,17,18,19]. The conventional physical detection of an impression, with trays and materials (alginates, silicones, polyethers), represents a substantial discomfort for patients [18,19,20]. In children and persons with a strong gag reflex [21], IOSs enable the impressions to be taken quickly, and no materials and trays are requested. Moreover, IOSs were shown to be as accurate as their conventional analogues for single teeth and short span bridges [21,22].

For these reasons, IOSs facilitate the the taking of impressions in children: easily, quickly and accurately. Moreover, for clinicians, optical impressions with IOS can resolve difficulties that arise with conventional impression detection, and especially with technically complex impressions [23,24]. IOSs are well tolerated by children since they do not require the use of conventional materials [22,23,24]

In this article, we present an innovative approach for treating children after root canals, using a digital workflow with IOS and computer-aided design/computer-assisted manufacturing (CAD/CAM) fabrication of the restoration. The protocol will be illustrated by a clinical case. The treatment approach presented here can facilitate and improve the care of children who undergo endodontic treatment.

## 2. Methods

**Figure 1:**
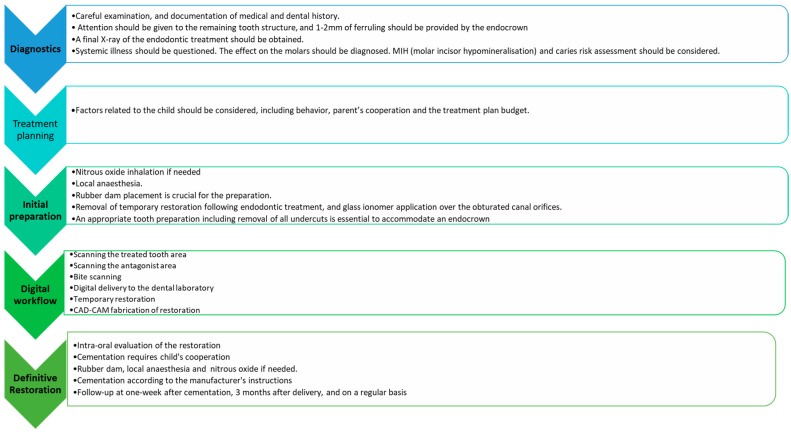
The suggested steps of the protocol of the endocrown treatment approach in endodontically-treated molars in children.

The suggested steps of the protocol of digital workflow with IOS and CAD/CAM fabrication of the restoration are described in Figure 1. The overall protocol involves five phases and a follow up.

The diagnostic phase consists of careful examination and documentation of the medical and dental history. Attention should be given to the remaining tooth structure. A final X-ray of the endodontic treatment should be obtained, as well as information on systemic illnesses. Diagnosing the effect on the risk of molar caries is crucial. Full attention should be paid to 1–2 mm of ferruling by the endocrown [1,12,13].

The treatment plan should address factors that are related to the child, his/her teeth and his/her family, such as the child’s behavior, the parents’ cooperation, compliance and the treatment plan budget.

In the initial preparation, nitrous oxide inhalation sedation should be considered for children who are not cooperative. Local anaesthesia must be administered. Rubber dam placement is necessary for the preparation. Temporary restorations must be removed following endodontic treatment, and glass ionomer must be applied over the obturated canal orifices. An appropriate tooth preparation, including removal of all undercuts or composite resin filling, is essential to accommodate an endocrown.

Digital workflow consists of IOS impressions, including scanning of the prepared tooth and its antagonist, scanning of the bite and CAD-CAM preparation of the restoration. Temporary restoration should be applied after the scanning.

Definitive restoration, including restoration bonding, is a crucial stage, especially in children. Since a rubber dam is essential for adequate cementation, effective local anaesthesia is necessary, together with the administration of nitrous oxide if required.

Follow up: the child should be invited for checkups one week after cementation, and 3 months later, and then on a regular basis, according to the caries risk assessment (Figure 1).

### 2.1. Compliance with Ethical Standards

The procedure described was performed in accordance with the ethical standards of the institutional research committee and with the 1964 Helsinki declaration and its later amendments.

### 2.2. Clinical Case

**Figure 2:**
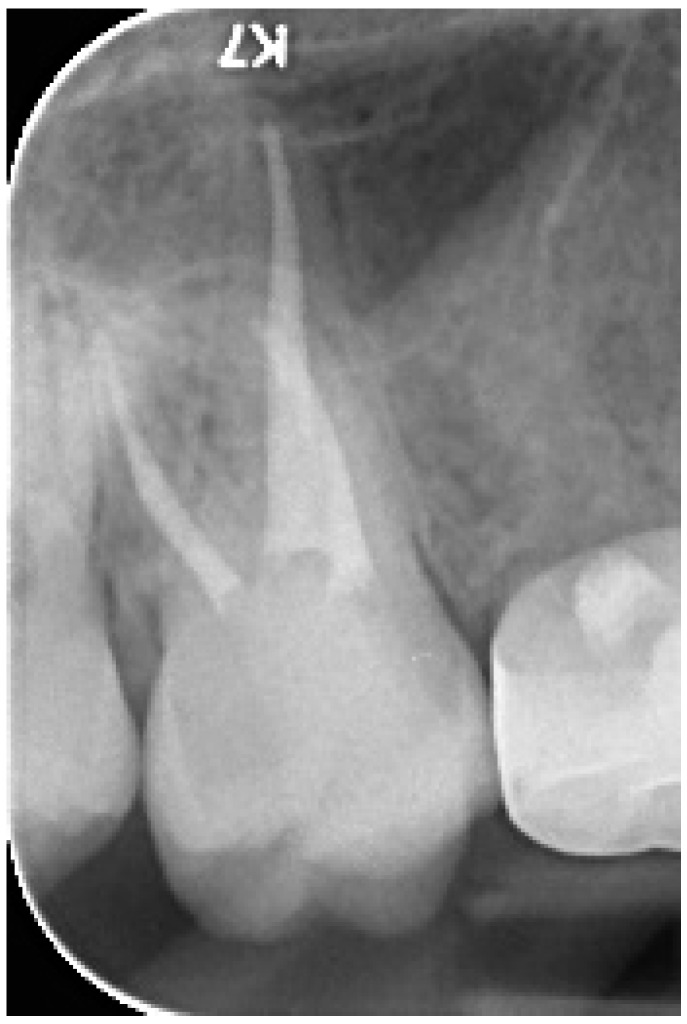
An X-ray after the completion of root canal treatment in the upper right first molar.

The following description of treatment for endocrown restoration illustrates the protocol of the treatment. An 11-year-old boy needed restoration after he underwent root canal treatment in his upper right first molar (#16) (Figure 2).

**Figure 3:**
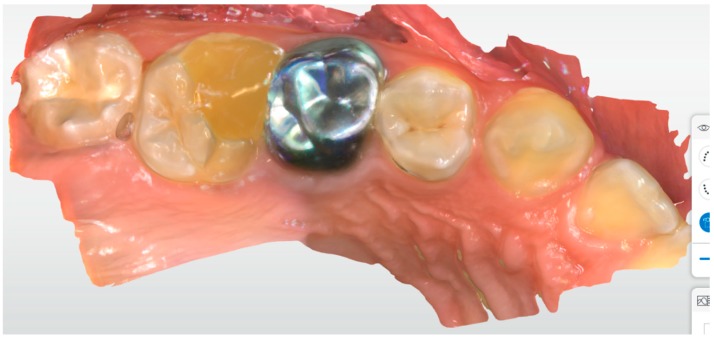
A scan of tooth #16 before tooth preparation.

His parents were interested in a definitive restoration. Full tooth coverage with an SSC was not considered an option by the parents, due to aesthetic reasons. Moreover, they wanted a less radical approach. The upper right first molar had a temporary restoration with well-preserved distal, buccal and lingual walls. The child’s cooperation was excellent and no inhalation sedation was needed (Figure 3).

**Figure 4:**
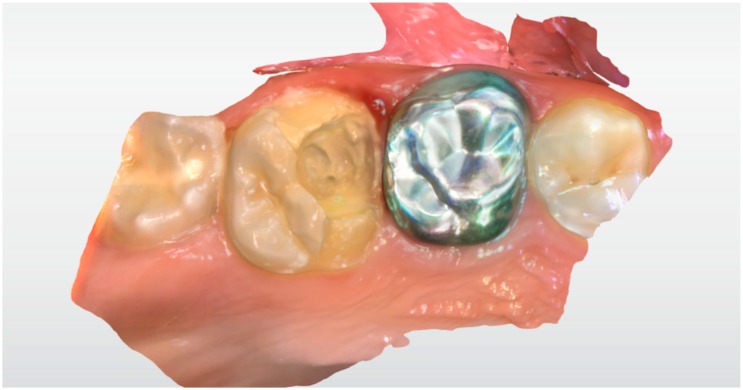
A scan of tooth #16 after tooth preparation.

**Figure 5:**
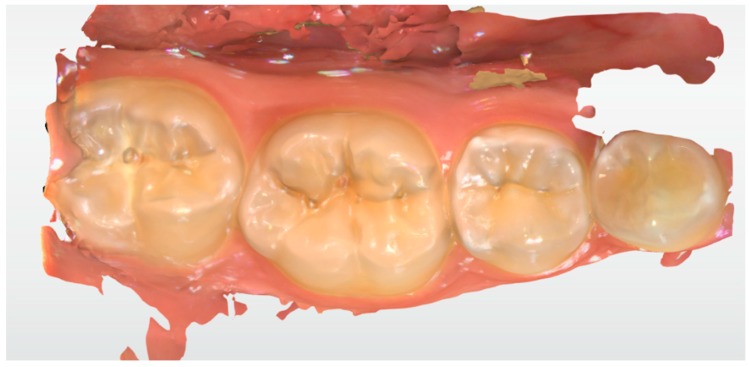
A scan of the antagonist jaw.

The child’s tooth was prepared to receive an endocrown restoration using local anaesthesia and a rubber dam. At the same appointment, the prepared tooth, the antagonist and the bite occlusion were scanned using IOS: Primescan connect® (Dentsply Sirona Dental Systems GmbH Bensheim, Germany), software connect version no. 5.1.0 (Dentsply Sirona Dental Systems GmbH Bensheim, Germany) (Figure 4 and Figure 5). The capture of the scan lasted for about 2 min and was very easy.

Telio® (Ivoclar-Vivadent, Schaan, Liechtenstein) was selected as temporary restoration material.

The child was scheduled one week later for cementation of the prepared lithium disilicate (LS2) glass-ceramic (IPS e.max Lithium Disilicate®, Ivoclar- Vivadent, Schaan, Liechtenstein ) CAD/CAM restoration.

**Figure 6:**
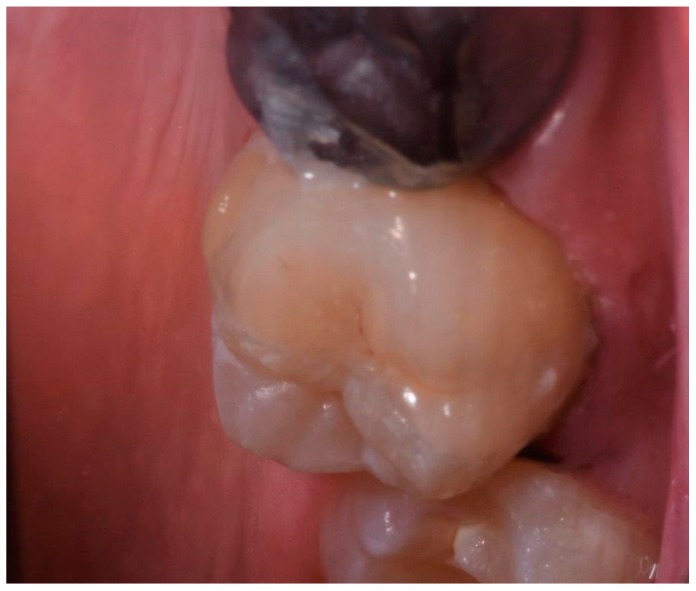
Cementation of restoration.

**Figure 7:**
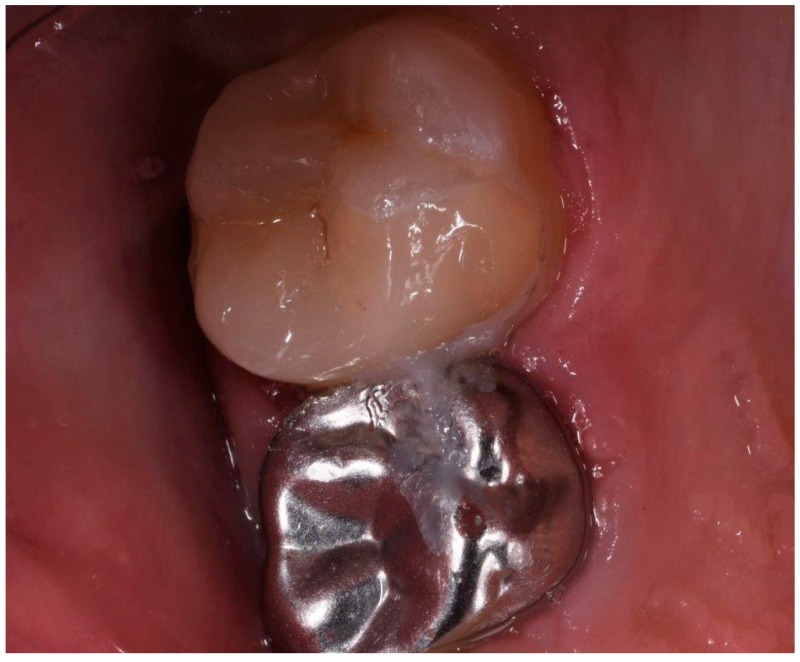
Cementation of restoration.

Local anaesthesia and a rubber dam were used. Variolink® (Ivoclar-Vivadent, Schaan Liechtenstein) was selected as the bonding agent (Figure 6 and Figure 7).

**Figure 8:**
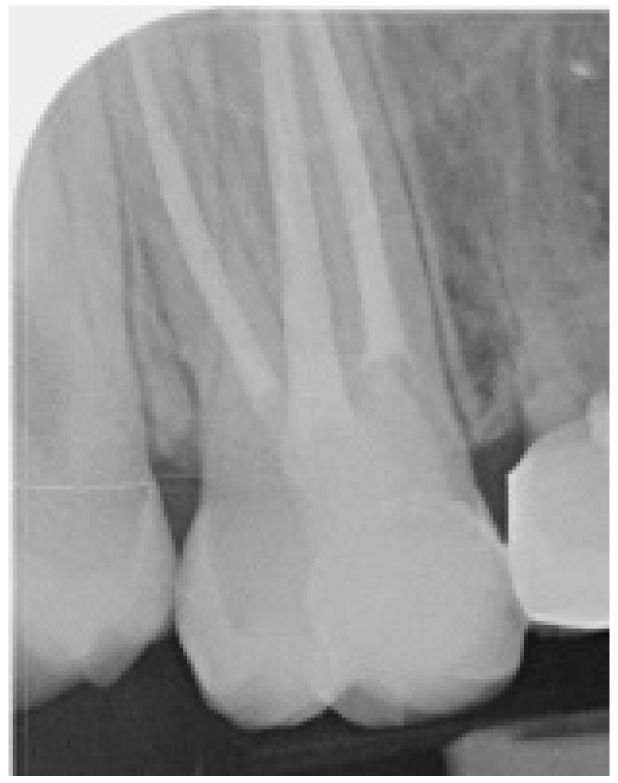
Final X-ray demonstrating marginal integrity and correct fit of the restoration.

One week later, at the follow-up appointment, the restoration was inspected clinically for the integrity of marginal fit and occlusion. An X-ray was obtained for the determination of marginal integrity and correct fit of the restoration (Figure 8).

Table 1 presents restorative options for endodontically-treated teeth. The table describes the decision-making considerations encountered by a paediatric dentist when dealing with the restoration of a child with root canal treatment. Below, we will address the advantages and disadvantages of the restorative options and discuss why the suggested treatment protocol is preferable, in our view, for endodontically-treated teeth in children.

## 3. Discussion

Endocrowns are rarely used as restorations in the paediatric population due to the lack of cooperation of children. This makes conventional impression-taking very challenging and, frequently, even impossible.

The introduction of restorative digitalization protocols, with IOS and CAD-CAM, to the prosthetic field of dentistry has opened a new venue for paediatric dentists and practitioners who treat young children. In this population, delivering the best restorative treatment for endodontically-treated permanent molars is challenging, due to the lack of cooperation during the conventional impression-taking procedure. To the authors’ best knowledge, the treatment approach presented in this article for children with endodontic treatment has not been previously described in the literature.

CAD/CAM components, with their adhesive technology afforded by the endocrown restoration, offers paediatric dentists an effective and conservative treatment option for the restoration of endodontically-treated teeth [1,12,13]. The endocrown has gained clinical acceptance for the restoration of endodontically-treated teeth in adults and can be easily used in children.

Mittal et al. [25] evaluated the clinical performance of indirect resin composite onlays (IRC onlay) compared to SSCs, as an aesthetic alternative for the rehabilitation of extensively carious primary molars. Each of 50 paediatric patients received IRC onlays or SSCs on extensively carious endodontically-treated primary molars. The cumulative survival rate of the IRC onlays compared to the SSCs was 82.9% versus 90.7% over a period of 36 months. Differences between the study groups in retention, marginal integrity, secondary caries, proximal contact, occlusion and gingival health were not statistically significant, as assessed at a number of time intervals. The IRC onlays required significantly less mean chair-side treatment time and were preferred by most parents and children.

Nagasiri et al. [26] explored the assumption that teeth are more prone to cracks after endodontic treatment and should, ideally, be crowned, especially posterior teeth. They evaluated the survival rate of endodontically-treated molars without crown coverage to identify associated factors. The overall survival rates of endodontically-treated molars without crowns at 1, 2 and 5 years were 96%, 88% and 36%, respectively. Survival increased as the amount of remaining coronal tooth structure increased. The five-year survival rate for molar teeth with maximum tooth structure remaining after endodontic treatment was 78%. The survival rate for restorations with direct composite was better than for restorations with amalgam with reinforced zinc oxide and better than for eugenol with polymethacrylate [26].

SSCs are the treatment of choice when teeth have moderate to severe hypoplasia and after root canal treatment [27,28,29]. The rationale for full coverage restoration with SSCs includes prevention of further tooth deterioration, the establishment of correct interproximal contacts and proper occlusal relationships, relatively low technique sensitivity and costs compared to cast restorations, and the minimal time required to prepare and insert [6,28,30]. If not adapted properly, however, SSCs may produce an open bite, gingivitis or both [21]. Properly placed, SSCs can preserve teeth with molar incisor hypomineralization until cast restorations are feasible [29,30]. However, the main disadvantages of SSCs are that they are not a definitive restoration and need replacement after the child grows. Moreover, the aesthetic appearance is generally not appealing to either children or their parents.

The use of IOSs has opened a new venue for restoring endodontically-treated teeth by reducing the challenge of a child’s behavior and enabling tooth structure preservation and long-lasting restoration. Digital dentistry has become more accessible and evidence-based in daily dental practice. According to the authors’ experience, digital workflow should be one of the preferred choices for the treatment of endodontically-treated molars, since the highly accurate scanning provides definitive restorations in young patients.

Partial and full coverage of indirect adhesive crowns and endocrowns should be considered for endodontically-treated molars in late mixed and permanent dentitions [28,29,30,31,32]. Such restorations are rarely used in young children due to placement difficulties associated with (1) short crowns, (2) long treatment time and high costs, and (3) the child’s limited cooperation [21,27]. However, the use of laboratory-fabricated crowns of cast gold, indirect composite and ceramic in 6- to 8-year-old children, was described as clinically effective over a 2- to 5-year follow-up [21].

Compared to SSCs, endocrown restorations require minimal tooth reduction, eliminate the need for post and core restorations, protect tooth structure, provide high strength for cuspal overlays, preserve tooth structure and maintain periodontal health due to their supragingival margins [30,31,32]. Others argue that the quality and longevity of cast adhesive copings and preformed SSCs are similar [6]. The decision to restore endodontically-treated molars with either indirect adhesive restoration or preformed SSCs should be based on the patient’s immediate and long-term needs, the child’s and parents’ cooperation, treatment costs, the clinician’s skills and the materials available.

## 4. Conclusions

The restoration of endodontically-treated teeth in children is always problematic for paediatric dentists. On one hand, interim SSC restoration is very unaesthetic and must be replaced at a later time. On the other hand, impression-taking in children is very difficult and requires the child’s cooperation. The suggested digital workflow presented here enables the clinician to provide a definitive and durable restoration solution in endodontically-treated molars in children.

## Figures and Tables

**Table 1:** Decision-making considerations regarding the restoration restorative options for endodontically-treated teeth in children.

Restorative Considerations	Composite Restoration	Full Coverage Stainless-Steel Crown	CAD/CAM Ceramic Endocrown
Effectiveness of restorative approach	++	+++	+++
Tooth preparation	++	+	++
Restoration strength (shear & bond)	++	+++	+++
Occlusal contact stability	++	+++	+++
Interproximal contact stability	++	+++	+++
Aesthetics	++	-	+++
Technique sensitivity	++	+++	++
Cost	++	++	+
Predictability	+	+++	+++
Need for definitive restoration	+	-	+++
Restoration longevity	++	+++	+++

+ Poor, ++ Fair, +++ Excellent.
